# Supplementary material for: Topological delocalization and tuning of surface channel separation in Bi2Se2Te Topological Insulator Thin films
Source: Sci Rep. 2017 Jul 7;7:4924. doi: 10.1038/s41598-017-04458-2 (PMC5501793; doi:10.1038/s41598-017-04458-2)
Supplement: Supplementary file 1 — Supplementary Information [file 41598_2017_4458_MOESM1_ESM.pdf]

Supplementary Information

Topological delocalization and tuning of surface channel separation in Bi_2Se_2Te Topological Insulator Thin films

R.K. Gopal, Sourabh Singh, Arpita Mandal, Jit Sarkar, Chiranjib Mitra

Indian Institute of Science Education & Research, Kolkata, India

The detailed study of the X-ray diffraction (XRD) had been carried out to verify the correct phase for at least 20 samples deposited by Pulse Laser Deposition (PLD) at different conditions. As it can be seen from the fig. S1 not all the BST phase related peaks can be found in all the XRD patterns. While depositing BST thin films one or two copies were always prepared for the different characterization procedures such as Scanning Electron Microscopy (SEM), Energy Dispersive X-ray (EDX) spectroscopy and Raman spectra.

Here we present detailed XRD analysis to get bulk insulating ordered phase of BST thin films deposited in different conditions. The substrate temperature was varied while keeping the laser energy density fixed at a value of 1.5 J/cm^2. However, the best XRD pattern matching with the ordered BST phase, where, Se atoms occupy central part of the QL is shown by the thin films deposited between 300ºC – 350ºC[1–3]. Annealing at slightly higher temperatures is always helpful to have a smoothened surface and to help reduce the defects in the bulk of the sample by arranging the Se/Te atoms at the preferred low energy locations in the QL. Hence, we annealed samples after the deposition at 30ºC above the deposition temperature.

BST and BTS like tetradymite belong to the rhombohedral crystal structure with space group of $R\bar{3}m$. These alloys and their chalcogen ordered form (Se atoms occupying the low energy preferential sites between Bi layers) and associated peaks with this ordered structure can be seen in fig. S2 (009, 107 and 0012)[4].

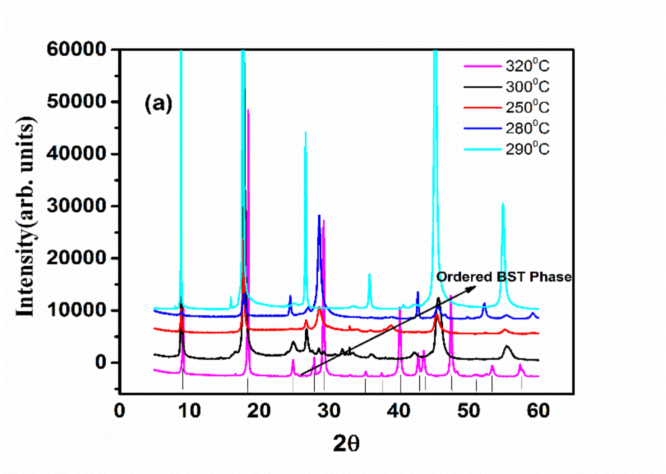

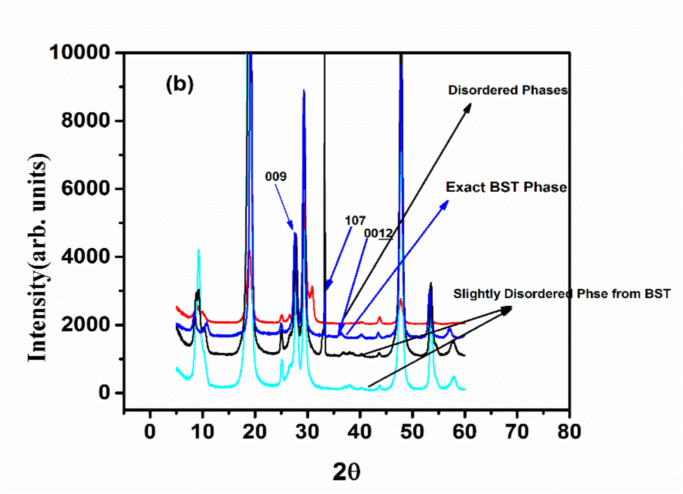

FIG. S1 (a) X – ray diffraction pattern of BST thin films deposited at different temperature at a fixed laser energy density of 1.5 J/cm^{2}. Thin films deposited at 320ºC are in good agreement with the ordered chalcogen layers in the TI QL. (b) Thin films deposited at different temperature and at a fixed laser energy density of 1.5 J/cm^{2} again the temperature window of getting ordered BST phase lies in the window of 300ºC – 350ºC.

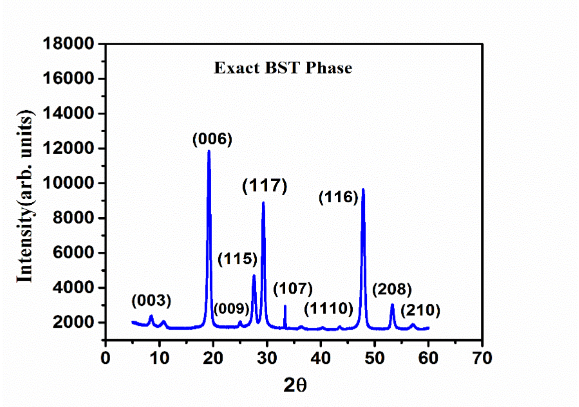

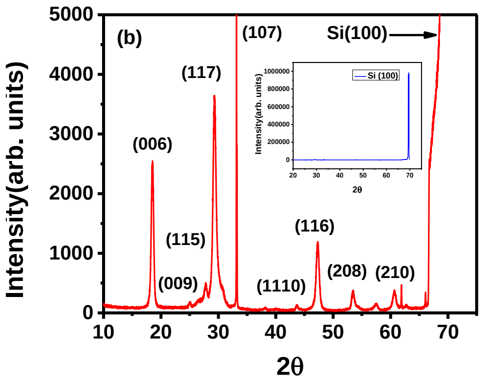

FIG. S2 (a) X – ray diffraction pattern of a BST thin film with ordered phase. (b) XRD pattern till 70° that includes the Si peak at ~ 69°. Though we have collected the XRD data till 70 degrees, we have shown it only up to 60 degrees as there is an enormous Si-substrate peak at 69 degrees which masks the sample peaks. However, despite processing the Si substrate in HF, this peak isn't affected indicating a good quality of the substrates even after HF treatment.

SEM and EDX of the BST thin films:

Scanning electron microscopy was performed on the BST thin film, with the energy dispersive X- ray spectra to determine the surface morphology and the grain size and composition of elements. It is shown in the EDX data of the films that films are not exactly Bi_2Se_2Te and there is a slight deficiency of Selenium for the B3 sample which is metallic in nature. Sample B1 and B2 which are insulating in nature have marginally higher Se content than Bi (see Table S1). The target of the BST was made a little Se rich (by alloying BS and BT in the weight ratio of (2.3-2.2/1) to maintain exact stoichiometry of the thin films. Even though in BTS[5,6] it is seen that slight Selenium vacancies lead to better insulating properties, in BST (in our case), slightly Se rich phase makes it insulating (B1&B2), but slight Se deficiency makes it metallic. The extra Selenium in B1 and B2 probably sit in the grain boundary or in the interstitial. Se vacancy makes the TIs electron doped and it is evident that the doping in B3 sample is significant as reflected by its high carrier density ($10^{20}/cm^3$).

(a)B1

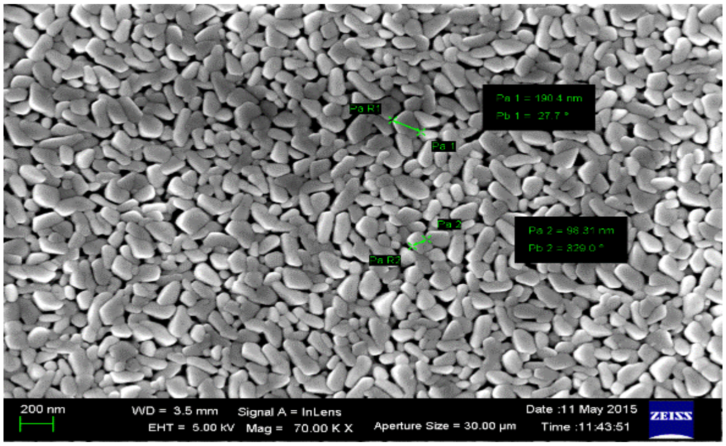

ELEMENT	WEIGHT (%)	ATOMIC (%)
Se	23.73	41.62
Te	18.60	20.18
Bi	57.66	38.20

(b)B2

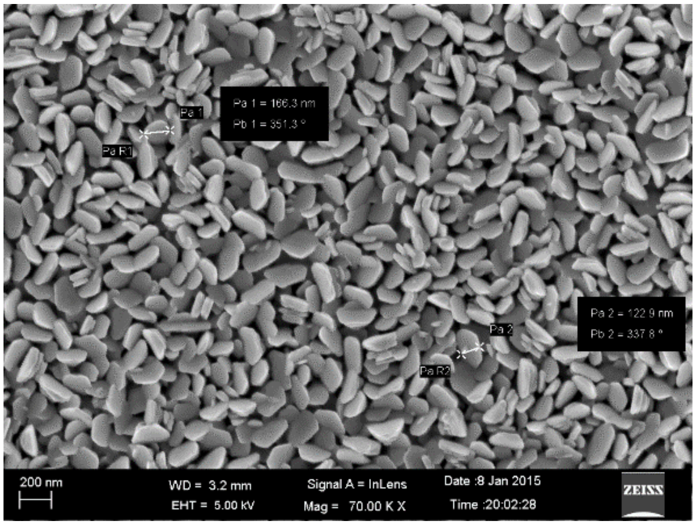

ELEMENT	WEIGHT (%)	ATOMIC (%)
Se	24.03	41.99
Te	18.62	20.13
Bi	57.36	37.87

(c)B3

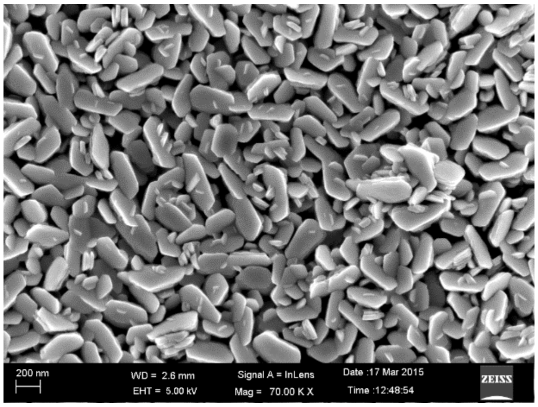

ELEMENT	WEIGHT (%)	ATOMIC (%)
Se	20.19	37.04
Te	17.24	19.58
Bi	62.57	43.38

FIG. S3 SEM images of (a) B1, (b) B2 and (c) B3 thin films and the respective EDX is shown here. The table shows the composition of our thin films. All the SEM images are taken in high magnification of 70 KX.

We did not anneal the metallic sample and as a result the Se/Te atoms do not occupy the preferred low energy configuration sites (random occupation in the chalcogen layer) in the quintuple layer and this results in Se vacancies in the crystals and Bi/Te antisite defects which generate bulk residual carriers making the same sample metallic in nature. On the other hand a controlled annealing in Ar atmosphere helps Se and Te atoms to diffuse in the preferred low energy sites in the middle of the quintuple layer, especially for the Se atoms, which results in a lesser vacancies and defects in the crystal and hence very less residual carriers. This results in a bulk insulating TI sample. Another reason for the reduced number of defect and vacancy generated carriers is the reduced grain boundary defects by annealing, as many adjacent/neighboring grains coalesce and nucleate making a single grain thus reducing the large number of defects in the grain boundary region.

Sample ID	Deposition Temp. (°C)	Annealing Temp. (°C)	Number of shots	Sample Type	Composition
B1	260	290	1500	Insulating	$Bi_{1.94}Se_{2.06}Te$
B2	260	290	5000	Insulating	$Bi_{1.92}Se_{2.08}Te$
B3	260	No annealing	1500	Metallic	$Bi_{2.10}Se_{1.90}Te$

Table S1. Sample deposition parameters of the three BST thin films studied.

Raman Spectrum Analysis:

BST or intermediate alloy solution has been analyzed in the past by using Raman spectroscopy and our thin films are in agreement to the results shown in that article and recent new studies[7–9]. The Raman spectra are shown in the fig. S4 for these thin films. As it can be seen from the spectrum all the films are of good crystallinity in nature exhibiting clear Raman peaks at 117.72 cm^{-1}, 140.19 cm^{-1} and 164.91cm^{-1} with corresponding modes E^2_g and A^1_g, with a slight deviation in the peak positions.

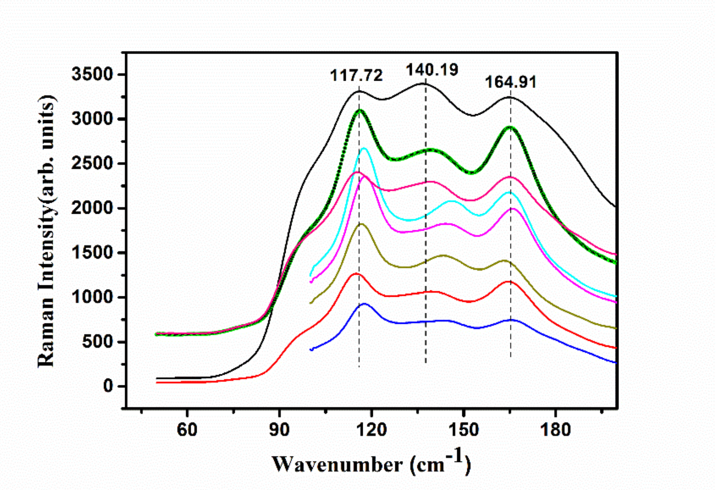

FIG. S4 Raman spectrum for the BST thin films deposited at different temperatures keeping laser fluence fixed at 1.5 J/cm^2. The dashed line corresponds to the three peaks for three Raman modes of BST (doubly degenerated E^2_g and A^1_g mode respectively).

***Comparison between BST and BS Raman Spectra*:**

Since BST has a close relationship with the BS in terms of crystal structure below we provide a comparison between the Raman peaks. It is quite evident that an extra vibration mode at 111.08cm^{-1} develops in comparison to the two characteristic BS Raman peaks. This peak is the manifestation of the presence of BT which has a peak centered at 100 cm^{-1}.

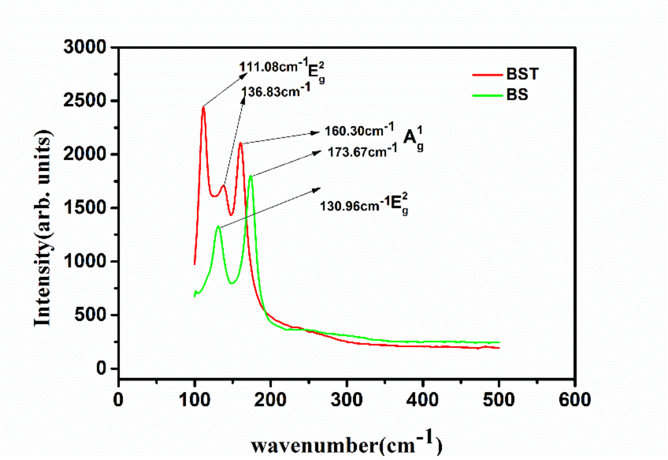

FIG. S5 Raman spectrum of BS and BST samples are shown in the figure. There is an extra peak in the Raman spectrum of BST.

BST thin films were grown on Si (001) substrates at different temperatures (250°C- 350°C). Substrate heating is an important parameter for epitaxial and ordered growth of thin films. The composition and quality of the film is affected by the reaction kinetics and the reaction rate of the reactant species. The substrate temperature controls both the reaction rate and its kinetics. We observed that samples prepared in 250°C – 300°C were better in comparison to the ones grown on high substrate temperature in terms of morphology, grain size and crystal structure. The substrates were cleaned by immersing them in 5% HF solution for 1 minute followed by ultrasound bath for 15 minutes in DI water. Prior to the growth substrates were degassed at an elevated temperature of 400^0C and subsequently flushed by inert gas (Argon) twice to get rid of impurities and oxide contaminations on the surface. Substrates were then cooled slowly to the deposition temperature. The ablation target material for BST thin films was prepared by grinding BS and BT materials in a stoichiometric ratio of 2:1 to get exact BST composition of thin films. The finely ground resultant material was pressed to form a pellet and subsequently it was then annealed at 400°C for 4hr in an inert (Argon) atmosphere followed by cooling it slowly for 4hrs.

Thin films of thicknesses nearly 150 nm and 500 nm were deposited at low repetition rate of 1Hz and at a laser fluence of 1.5 J/cm^2. After deposition, the films were annealed at elevated temperatures (30°C higher than deposition temperature) in Argon atmosphere for 1 hour and cooled naturally to room temperature in the same atmosphere. As opposed to single crystals PLD grown thin films have lower mobility due to the presence of disorder and grain boundaries.

Annealing:

Due to the non-equilibrium process of sample formation in the PLD technique, as grown thin films are in a state of instability. The presence of inhomogeneties, tensile stress and other undesirable defects renders the film in a metastable phase. It is essential to get rid of these unwanted defects and bring the film to be in a state of stabilization. Annealing is an effective method to stabilize these thin films. Heat treatment procedures after sample formation results in producing bulk insulating films (B1 and B2). Thus, annealing becomes an effective tool to alter the amount of defects in a sample. This in turn renders it the capacity to drive the chemical potential across the band gap.

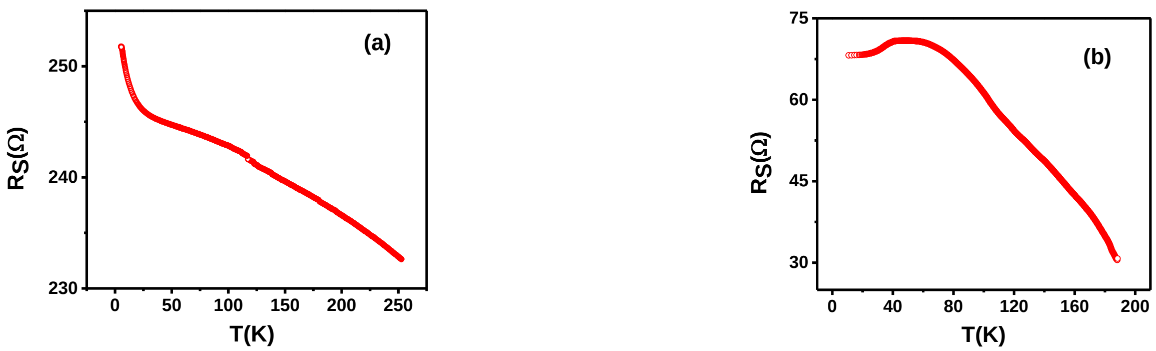

Figure S6. (a) Resistance vs. Temperature of BST sample annealed at 300°C. (b) Resistance Temperature profile of BST sample annealed at 330°C.

Figure S6 shows two more BST thin films of the same series. These films were annealed at 300 and 330 degree C respectively. Increasing the annealing temperature and annealing time results in increase of the grain size. But there is a threshold temperature above which increasing the annealing temperature results in aggregation of the grain boundaries.

TRANSPORT MEASUREMENT

Transport measurements on thin films were carried out in a cryogen free magnet (Cryogenic, U.K.) in the temperature range of 1.6 K to 300 K and a maximum magnetic field of 8 Tesla. Before placing the sample inside the cryogenic system a Hall bar pattern was fabricated using optical lithography technique. Hall and magnetoresistance measurements were performed simultaneously and using one carrier model the carrier concentration was obtained from the Hall

voltage. The variation of electron concentration with temperature for sample B1 (insulating) and B3 (metallic) is shown in figure S7.

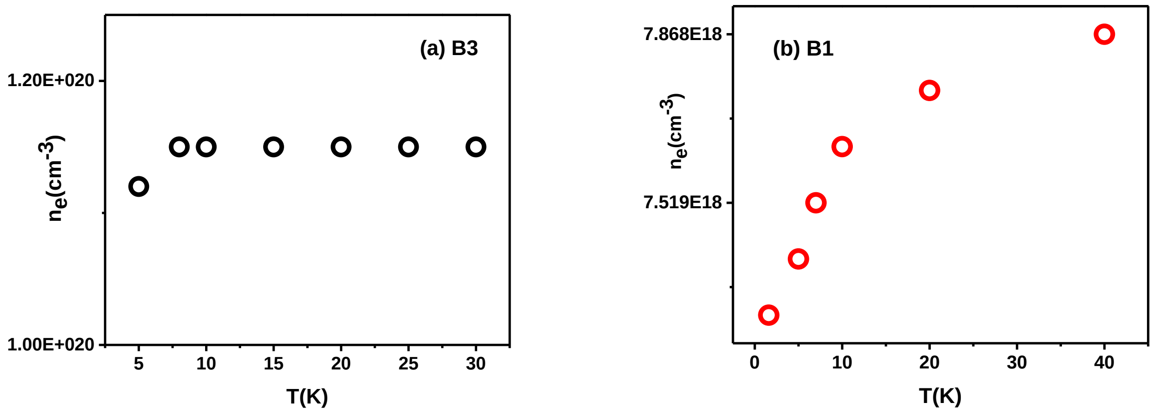

Figure S7 (a) Carrier concentration as a function of temperature in case of metallic sample B3. (b) Variation of carrier concentration with increasing temperature for the bulk insulating sample B1.

The resistance of thin films is called sheet resistance (R_S). From the sheet resistance the change in sheet conductance is obtained by

$$\Delta G_S = [R_S(B) - R_S(0)]/[R_s(0)^2]$$

This ΔG_S is fitted with HLN equation and the fitting parameters are obtained from it. In the low temperature regime the conductance variation with temperature deviates from the Arrhenius behavior T^{-1} owing to the dominance of surface states in the conductance mechanism. But in the intermediate and high temperature regime the bulk dominates the proceedings. In the intermediate temperature regime the R-T curve exhibits Variable range hopping behavior (VRH) as seen earlier in disordered semiconductors[10] (figure S8). The presence of electron-electron interaction results in a cross-over from Mott VRH to Efros-Shklovski VRH behavior. This cross over is shown by the extended dashed lines in Fig. S8.

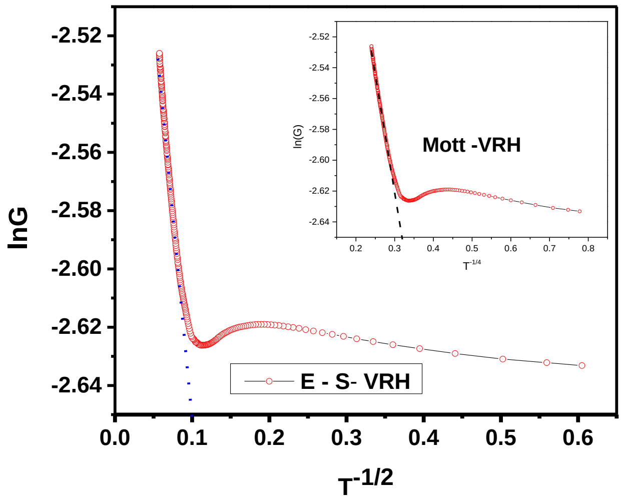

Figure S8. lnG vs. $T^{-1/2}$ to demonstrate the Efros –Shklovski (ES) variable range hopping (VRH). The straight line fit depicts the temperature regime in which the system follows ES behavior. The inset shows the regime in which the sample follows Mott VRH.

Weak Antilocalization (WAL) and Electron-Electron interaction (EEI) both invoke a logarithmic temperature dependent correction to the conductance in the low temperature regime with different signs[11,12]. A perpendicular magnetic field is applied to destroy WAL thus only EEI survives which is independent of the magnetic field. For B =1T and a reference temperature of 25K which is the temperature at which there is an upturn in the R-T behavior for sample B1. We utilize the formulation as given in reference 12 and obtain β = 3.82. This yields the screening factor F= -3.76, which is a huge deviation from the theoretically predicted values[11] thus enunciating the fact that the upturn in R-T is not only due to EEI but it also involves freezing of the bulk carriers**.** This result in itself signifies the surface to bulk coupling in our TI thin films and its signature is observed in the R-T curve itself. A similar result is obtained for the sample B2.

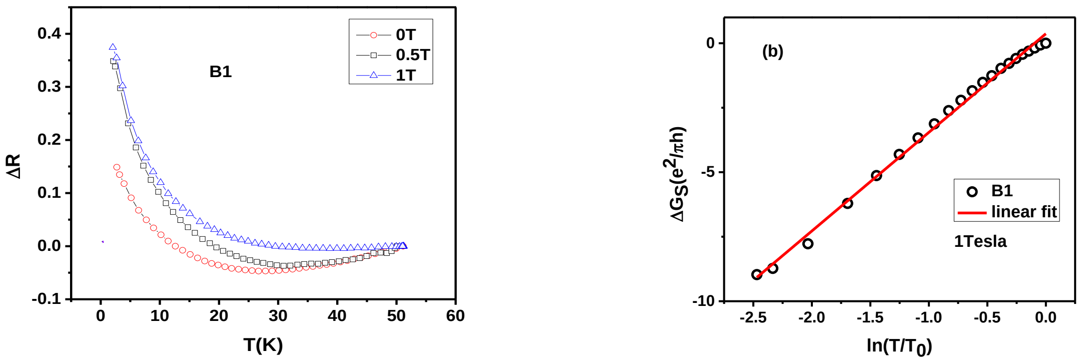

Figure S9 (a). Application of perpendicular magnetic field which results in destruction of WAL and thus the slope increases as the magnitude of field is increased. (b) Sheet conductance as a function of logarithm of temperature for sample B1.

<u>High Field fitting by Modified HLN equation:</u>

In our case, there is a gap of at least half an hour between sample growth of PLD deposition and sample processing it takes at least half an hour, before one can load the sample in the cryostat, which is sufficient for 2DEG to form on its surface. However, we do not know whether these additional near surface bands are Rashba split or not, as found experimentally in various cases by ARPES. Moreover, despite displaying insulating character (see fig.1 in manuscript), it is evident from the Hall data that there is still sizable bulk electron density present in these thin films even at the lowest of the temperature achieved (at 2K, $n_e = 7\times 10^{18}/cm^3$), however the bulk electrons are on the verse of localization as seen from VRH behavior. To account for such additional trivial electrons on surface and bulk electrons effects on MR we fit high field data (8T) with the modified HLN equation, with the assumption that these extra electrons will contribute in MR in quadratic form, also known as the Kohler form. This modified HLN equation can be written as:

$$\Delta G_S = -\frac{\alpha e^2}{2\pi^2\hbar}\left[\Psi\left(\frac{B\phi}{B}+\frac{1}{2}\right)-\ln\left(\frac{B\phi}{B}\right)\right]+\lambda B^2 \qquad \text{(S1)}$$

Where all the parameters are same as before in equation (4) and the Kohler's term lB^2 captures the possible contribution from the bulk electrons in MR. On fitting MR with the modified HLN equation in the high field limit, i.e. up to 8T, the value of α remains nearly "-1" (for the 2 K

data), which is consistent with the value for two surfaces. It signifies the presence of the two conduction decoupled channels in these thin films.

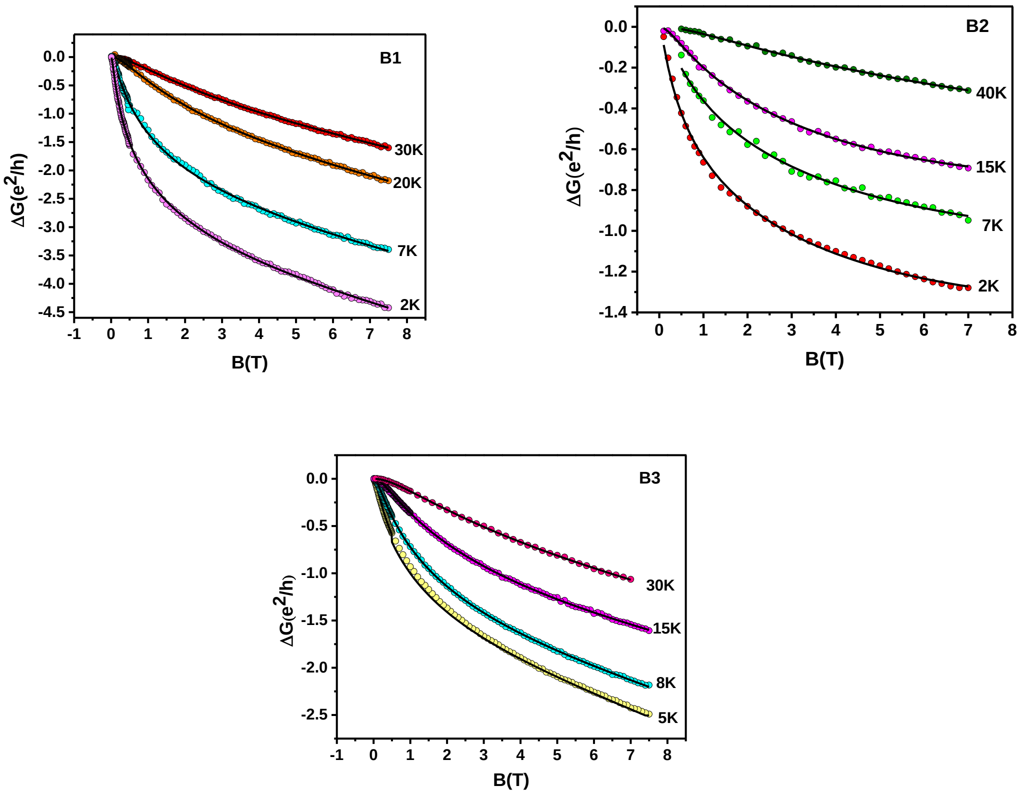

Figure S10. The fitted high field MC with modified HLN equation (S1) for the thin films B1, B2 and B3.

<u>**References**</u>:

1. Cao, H. *et al.* Controlling and distinguishing electronic transport of topological and trivial surface states in a topological insulator. arXiv: 1409.3217 (2014)

2. Jauregui, L. A. *et al.* Gate Tunable Relativistic Mass and Berry's phase in Topological Insulator Nanoribbon Field Effect Devices. *Sci. Rep.* **5,** 8452 (2015).

3. Ren, Z., Taskin, A. A., Sasaki, S., Segawa, K. & Ando, Y. Large bulk resistivity and surface quantum oscillations in the topological insulator Bi_2Te_2Se *Phys. Rev. B* **82,** 241306 (2010).

4. Ren, Z., Taskin, A. A., Sasaki, S., Segawa, K. & Ando, Y. Optimizing $Bi_{2-x}Sb_xTe_{3-y}Se_y$ solid solutions to approach the intrinsic topological insulator regime. *Phys. Rev. B* **84,** 165311 (2011).

5. Irfan, B. & Chatterjee, R. Magneto-transport studies on $Bi_2Te_{2+x}Se_{1-x}$ (x = 0.05 and 0.10) topological insulators. *AIP Adv.* **6,** 95215 (2016).

6. Kushwaha, S. K. *et al.* Sn-doped $Bi_{1.1}Sb_{0.9}Te_2S$ bulk crystal topological insulator with excellent properties. *Nat. Commun.* **7,** 11456 (2016).

7. Nakajima, S. The crystal structure of $Bi_2Te_{3-x}Se_x$. *J. Phys. Chem. Solids* **24,** 479–485 (1963).

8. Chis, V. *et al.* Vibrations in binary and ternary topological insulators: First-principles calculations and Raman spectroscopy measurements. *Phys. Rev. B* **86,** 174304 (2012).

9. Schönherr, P. *et al.* Vapour-liquid-solid growth of ternary Bi_2Se_2Te nanowires. *Nanoscale Res. Lett.* **9,** 127 (2014).

10. Ren, Z., Taskin, A. A., Sasaki, S., Segawa, K. & Ando, Y. Fermi level tuning and a large activation gap achieved in the topological insulator Bi_2Te_2Se by Sn doping. *Phys. Rev. B* **85,** 155301 (2012).

11. Lee, P. a. Disordered electronic systems. *Rev. Mod. Phys.* **57,** 287–337 (1985).

12. Chiu, S.-P. & Lin, J.-J. Weak antilocalization in topological insulator Bi_2Te_3 microflakes. *Phys. Rev. B* **87,** 035122 (2013).
